# Serum KL-6 and SP-D: Markers of Lung Function in Autoimmune-Related Interstitial Lung Diseases

**DOI:** 10.3390/ijms26031091

**Published:** 2025-01-27

**Authors:** Ewa Miądlikowska, Joanna Miłkowska-Dymanowska, Adam Jerzy Białas, Joanna Samanta Makowska, Anna Lewandowska-Polak, Anna Puła, Anna Kumor-Kisielewska, Wojciech Jerzy Piotrowski

**Affiliations:** 1 Department of Pneumology, Medical University of Lodz, 90-419 Lodz, Poland; ewa.miadlikowska@umed.lodz.pl (E.M.); joanna.milkowska-dymanowska@umed.lodz.pl (J.M.-D.); adam.bialas@umed.lodz.pl (A.J.B.); anna.kumor@umed.lodz.pl (A.K.-K.); 2 Department of Pulmonary Rehabilitation, Regional Medical Center for Lung Diseases and Rehabilitation, Blessed Rafal Chylinski Memorial Hospital for Lung Diseases, 91-520 Lodz, Poland; 3 Department of Rheumatology, Medical University of Lodz, Zeromskiego 113, 90-549 Lodz, Poland; joanna.makowska@umed.lodz.pl (J.S.M.); anna.lewandowska-polak@umed.lodz.pl (A.L.-P.); 4 Department of Hematology, Medical University of Lodz, 93-510 Lodz, Poland; anna.pula@umed.lodz.pl; 5 Section of Hematology/Oncology, University of Chicago, 5841 S. Maryland Ave, MC 2115, Chicago, IL 60637-1470, USA

**Keywords:** interstitial pneumonia with autoimmune features, connective tissue disease, biomarker, IPAF, CTD, KL-6, SP-D, TGF-β1

## Abstract

This study evaluates the usefulness of serum KL-6, SP-D and TGF-β1 levels in assessing lung impairment and predicting interstitial lung disease (ILD) short-term progression in patients with interstitial pneumonia with autoimmune features (IPAF). A total of 24 patients with IPAF, 21 with connective tissue disease-associated ILD (CTD-ILD) and 23 with CTD without ILD were followed for 1 year. Serum levels of KL-6, SP-D and TGF-β1 were measured and their associations with disease severity and progression were analysed. KL-6, SP-D and TGF-β1 levels were significantly higher in IPAF and CTD-ILD patients compared to CTD without ILD (*p* < 0.0001, *p* = 0.0005 and *p* = 0.0001, respectively). KL-6 (r = 0.45, *p* = 0.002) and SP-D (r = 0.35, *p* = 0.02) levels correlated with lung involvement in HRCT in the ILD group. In IPAF, KL-6 levels correlated with pulmonary function tests (FVC%, TLCO%, and 6MWD) and SpO2, while SP-D correlated with 6MWD and SpO2. In CTD-ILD, KL-6 and SP-D levels were positively correlated with BAL cell count (KL-6: r = 0.58, *p* = 0.04; SP-D: r = 0.63, and *p* = 0.02). KL-6 also showed a negative correlation with the time since symptom onset (r = −0.51, *p* = 0.02). No significant associations were found between the baseline biomarker levels and ILD progression risk. KL-6 and SP-D may serve as potential biomarkers for assessing lung impairment in IPAF, though their predictive value for short-term prognosis remains uncertain.

## 1. Introduction

Interstitial pneumonia with autoimmune features (IPAF) is a term established by the American Thoracic Society and European Respiratory Society (ATS/ERS) in 2015 to describe a specific subtype of interstitial lung disease (ILD) [1]. Patients who meet these criteria exhibit certain features suggestive of an underlying autoimmune process, but they do not fulfil the criteria for any known connective tissue disease (CTD). Although it has been nearly a decade since its introduction, our understanding of this group of patients remains limited, primarily due to its heterogeneity [2].

The criteria adopted by ATS/ERS are widely debated. Patients with myositis-specific antibodies (MSA) are proposed to be excluded from IPAF due to distinct disease behaviour and treatment responses. Expanding the criteria to include features like sicca symptoms, ANCA, or UIP pattern on HRCT is also under discussion [3].

IPAF patients occupy an intermediate spectrum between CTDs and idiopathic pulmonary fibrosis (IPF). While IPF is a relatively homogeneous disease focused on lung fibrosis, the aetiology of ILD in CTD is complex, and it remains unknown as to why only some CTD patients develop ILD. Factors potentially driving CTD-ILD pathogenesis include inflammation, genetics, and environmental influence [4,5].

In both IPAF and CTD-ILD, patients exhibit similar clinical symptoms, comparable radiological patterns in high-resolution computed tomography (HRCT), findings in histopathological examinations, and lung impairments in pulmonary function tests (PFTs).

Recently, there has been a growing effort to identify tools that can precisely and non-invasively differentiate ILD subtypes or identify CTD patients at increased risk of developing or progressing ILD. Biomarkers, particularly molecules circulating in peripheral blood, are at the forefront of this research. Traditional ILD monitoring relies on spirometry, the lung transfer factor for carbon monoxide (TLCO), and the 6 min walk test (6MWT), which depend on the patient’s condition and cooperation. Additionally, HRCT scans, while informative, expose patients to radiation. For these reasons, an ongoing search for biomarkers can provide a simple, real-time assessment of whether a patient is experiencing the progression of interstitial lung disease. For IPAF patients, diagnosing ILD is challenging, as it requires excluding other lung diseases, often through invasive tests. Biomarkers could offer a safer, more practical option for assessment, especially when PFTs are difficult or impossible to perform [5,6,7,8].

Most studies on biomarkers in IPAF were conducted by Asian researchers, highlighting the need for studies involving Caucasian populations, as molecular measurement results can vary across different racial groups [9,10,11].

In this study, we aimed to answer whether there are serum biomarkers of lung fibrosis in IPAF that can be used in everyday clinical practice and whether their usefulness differs in patients with CTD. The biomarkers we focused on were transforming growth factor β1 (TGF-β1)—which promotes fibroblast differentiation into myofibroblasts that produce collagen and extracellular matrix components—and biomarkers of alveolar epithelial cell damage and dysfunction: Krebs von den Lungen-6 (KL-6) and surfactant protein-D (SP-D) [12]. The study aimed to determine whether KL-6, SP-D, and TGF-β1 can serve as reliable biomarkers for lung fibrosis in IPAF and CTD-ILD, assess their predictive value in evaluating the short-term progression of pulmonary fibrosis, and explore their associations with the clinical, functional, and radiological features of established predictive significance.

## 2. Results

### 2.1. Characteristics of Study Population

A total of 68 participants were included in the study: 24 patients with IPAF, 21 with CTD-ILD [systemic sclerosis (SSc): 13, idiopathic inflammatory myopathies (IIM): 4, rheumatoid arthritis (RA): 2, systemic lupus erythematosus (SLE): 1, the overlap of RA and Sjögren’s syndrome (SS): 1], and 23 CTD without ILD (SSc: 8, SS: 5, SLE: 3, RA: 3, IIM: 3, overlap of SS, and IIM: 1). All patients were Caucasian.

The detailed demographic and clinical characteristics of the participants are outlined in Table 1.

During a one-year follow-up of patients with IPAF, three of them converted into CTD-ILD (one SS, one SSc, and one IIM). One patient with IPAF and one with CTD-ILD (SSc) died. One SSc-ILD patient underwent a lung transplantation.

### 2.2. Biomarker Levels by Patient Group

**Figure 1:**
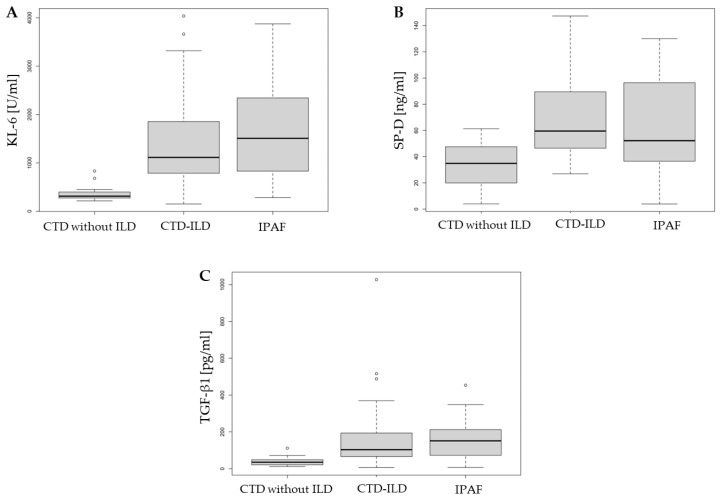
Comparison of serum biomarkers: (**A**) KL-6, (**B**) SP-D, and (**C**) TGF-β1 in IPAF, CTD-ILD, and CTD without ILD. IPAF—interstitial pneumonia with autoimmune features, CTD—connective tissue disease, ILD—interstitial lung disease, KL-6—Krebs von den Lungen-6, SP-D—surfactant protein D, and TGF-β1—transforming growth factor β1.

Significant differences were observed in the concentrations of KL-6, SP-D, and TGF-β1 among the IPAF, CTD-ILD, and CTD without ILD groups. In all cases, biomarker levels were significantly lower in the CTD without ILD group compared to both the IPAF and CTD-ILD groups. However, no significant differences were found in serum biomarker levels between the IPAF and CTD-ILD groups (Table 2, Figure 1).

Additionally, the correlations between the concentrations of the biomarkers and patients’ age, gender, BMI, presence of hypertension, or diabetes, or smoking history were analysed. However, no significant correlations or trends were identified.

### 2.3. Relationship Between Biomarker Levels and Quantitive Lung Involvement in HRCT

**Figure 2:**
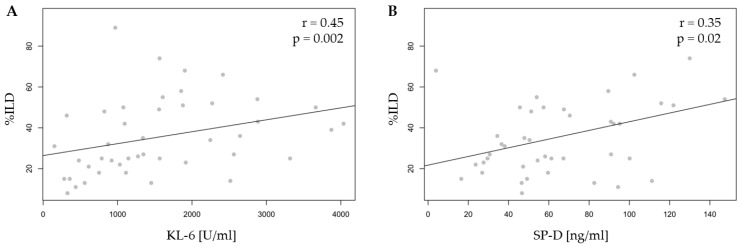
The correlation between serum biomarkers: (**A**) KL-6 and (**B**) SP-D and quantitative lung involvement (%ILD) in patients with IPAF and CTD-ILD, collectively assessed as one-group autoimmune-related ILD. KL-6—Krebs von den Lungen-6, SP-D—surfactant protein D, and ILD—interstitial lung disease.

In patients with ILD (IPAF and CTD-ILD combined), serum levels of KL-6 (r = 0.45, *p* = 0.002) and SP-D (r = 0.35, *p* = 0.02) showed a positive correlation with quantitative ILD assessment on HRCT (Figure 2). No such relationship was observed for TGF-β1. Furthermore, no correlation was found between the levels of any biomarker and the radiological pattern.

### 2.4. Correlations Between KL-6, SP-D, and TGF-β1 Levels and Lung Function at Baseline or Changes in PFTs During Follow-Up Across Different Groups of Patients

#### 2.4.1. IPAF

**Figure 3:**
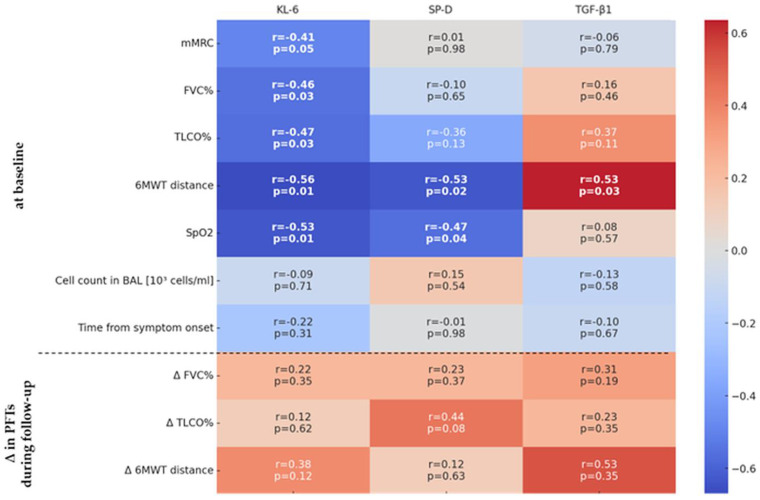
The correlation heatmaps between serum biomarkers KL6, SP-D, TGF-β1 and lung function or clinical indicators in the IPAF group. The heatmap colour corresponds to the r-value (−0.6 to +0.6). The upper row in each table square denotes the r-value of Spearman’s rank correlation, and the lower row denotes its *p*-value. The bolded values are statistically significant. IPAF—interstitial pneumonia with autoimmune features, mMRC—Modified Medical Research Council, FVC—forced vital capacity, TLCO—lung transfer factor for carbon monoxide adjusted for haemoglobin, 6MWT—six-minute walk test, SpO2—peripheral capillary oxygen saturation, as measured by pulse oximetry, BAL—bronchoalveolar lavage, and PFT—pulmonary function test.

**Figure 4:**
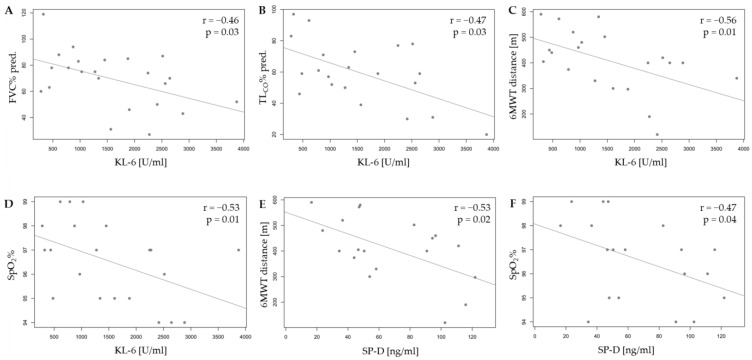
The correlation in the IPAF group between KL-6 level and FVC% predicted (**A**), TLCO% predicted (**B**), 6MWT distance (**C**), SpO2% (**D**) the SP-D level and 6MWT distance (**E**), and SpO2% (**F**). KL-6—Krebs von den Lungen-6, SP-D—surfactant protein D, FVC—forced vital capacity, TLCO—lung transfer factor for carbon monoxide adjusted for haemoglobin, 6MWT—six-minute walk test, and SpO2—peripheral capillary oxygen saturation, as measured by pulse oximetry.

We observed a negative correlation between KL-6 levels and all pulmonary function tests (PFTs) at baseline in patients with IPAF: FVC% (r = −0.46, *p* = 0.03), TLCO% (r = −0.47, *p* = 0.03), 6MWT distance (r = −0.56, *p* < 0.01), and SpO2 (r = −0.53, *p* = 0.01). SP-D levels inversely correlated with the 6 min walk test (6MWT) distance (r = −0.53, *p* = 0.02) and SpO2 (r = −0.47, *p* = 0.04). No significant associations were found between biomarker levels and changes in PFT results over the one-year observation period, except for the observed effect of baseline TGF-β1 levels on changes in 6MWT distance (r = 0.53, *p* = 0.03) (Figure 3 and Figure 4).

#### 2.4.2. CTD-ILD

In the CTD-ILD group, the concentration of KL-6 (r = 0.58, *p* = 0.04) and SP-D (r = 0.63, *p* = 0.02) correlated positively with the cell count in BAL, while a negative correlation was noted between KL-6 and the time from symptom onset (r = −0.51, *p* = 0.02). A positive correlation between KL-6 concentration and Δ 6MWT distance over the one-year period was also found (r = 0.74, *p* < 0.01) (Supplementary Figure S1). 

#### 2.4.3. IPAF + CTD-ILD

**Figure 5:**
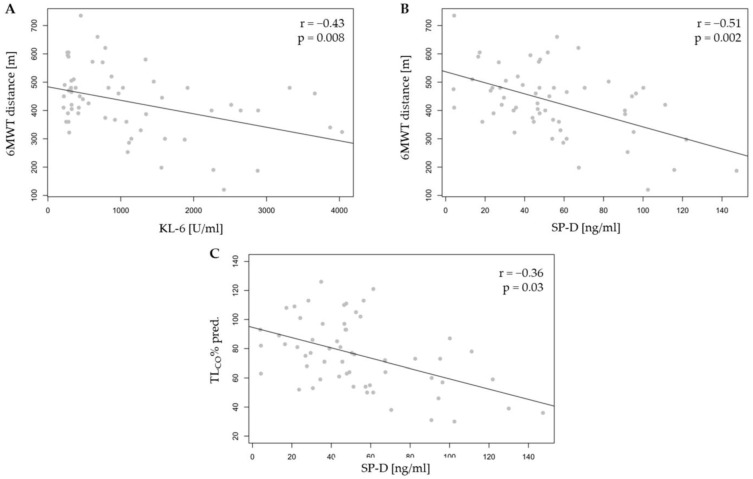
The correlation in the autoimmune-related ILD group (IPAF and CTD-ILD combined) between (**A**) KL-6 level and 6MWT distance, (**B**) SP-D and 6MWT distance, and (**C**) SP-D and TLCO%. KL-6—Krebs von den Lungen-6, SP-D—surfactant protein D, 6MWT—six-minute walk test, and TLCO—lung transfer factor for carbon monoxide adjusted for haemoglobin.

Correlation analysis in the whole autoimmune-related ILD group (IPAF and CTD-ILD combined) revealed a negative relationship between 6MWT distance and the concentrations of KL-6 (r = −0.43, *p* = 0.01) and SP-D (r = −0.51, *p* = 0.01). We also observed a negative correlation between TLCO% and SP-D (r = −0.36, *p* = 0.03) and a positive correlation between TLCO% and TGF-β1 (r = 0.34, *p* = 0.04). Additionally, a positive correlation between KL-6 levels and Δ 6MWT distance (r = 0.49, *p* < 0.01) was noted. The detailed results are shown in Supplementary Figure S2 and Figure 5.

### 2.5. Baseline Biomarkers and Progression Risk

In our study, we analysed the entire group of patients categorised as autoimmune-induced ILD (IPAF and CTD-ILD). We performed both univariate and multivariate logistic regression analyses, which showed that baseline serum levels of the biomarkers KL-6, SP-D, and TGF-β1 did not influence the risk of short-term progression, as defined by the four criteria analysed separately: PPF (2022 ATS/ERS/JRS/ALAT guidelines), the PF-ILD INBUILD criteria, Polish Respiratory Society guidelines, and FVC decline ≤10%.

## 3. Discussion

In our study, we evaluated the levels of KL-6, SP-D, and TGF-β1 in three groups of patients: IPAF, CTD-ILD, and CTD without ILD. To the best of our knowledge, this is the first study to compare these biomarkers among these groups. Based on our findings, we suggest that measuring biomarkers—particularly KL-6 and SP-D—could serve as an additional tool in clinical assessment in the future.

The findings of the study indicate that in patients with autoimmune-related ILD (IPAF and CTD-ILD) the levels of KL-6, SP-D, and TFG-β were higher than in patients with CTD without ILD. These results align with those presented in the literature. Previous studies have reported that elevated serum KL-6 levels were higher in patients with IPAF than those with non-fibrotic lung disease [9] or normal controls [10]. Zhou, A. et al. showed also that patients with CTD-ILD had a higher concentration of KL-6 in their blood than those without ILD [11]. Additionally, SP-D serum levels were higher in the CTD-ILD group compared to patients with CTD without ILD and healthy volunteers [13]. To date, there are no studies evaluating the serum TGF-β1 levels in patients with IPAF. Due to the lack of significant differences between the KL-6, SP-D, and TGF-β1 levels in our study, these molecules should not be considered tools for distinguishing between IPAF and CTD-ILD. However, to the authors’ knowledge, this is the first study comparing KL-6, SP-D, and TFG-β concentrations among the IPAF group vs. CTD-ILD and CTD without ILD. To date, there are no studies evaluating the serum TGF-β1 levels in patients with IPAF.

It is worth highlighting the significant correlations observed between serum biomarker levels and the extent of lung involvement in HRCT in patients with autoimmune-related ILD (IPAF and CTD-ILD). The levels of both KL-6 and SP-D were positively correlated with the percentage of interstitial lung disease (%ILD). However, this correlation was not observed for TGF-β1. Additionally, no correlation was found between the concentrations of these biomarkers and any specific radiological patterns. The findings of our study are confirmed by the research of Xue et al., who reported a positive correlation between KL-6 serum levels and HRCT score in IPAF patients [10]. Similar results were presented in the study by Ma, H. et al., who used CTD-ILD patients as the study group [14]. SP-D has not yet been evaluated in the IPAF group, but a correlation between the level of this biomarker and semi-quantitative scores for the degree of ILD has been previously described [13]. However, the methodology of quantitative assessment adopted in the cited studies differs from that adopted in our study.

These findings may have clinical implications in the future. HRCT is currently a precise tool for diagnosing and monitoring interstitial lung diseases, but its use involves radiation exposure. The possibility of using biomarkers, with strong evidence of their usefulness, could potentially reduce the frequency of imaging studies.

One key aspect of our study was the analysis of the utility of biomarkers as tools reflecting the degree of lung damage in three groups of patients: IPAF, CTD-ILD, and autoimmune-related ILD (IPAF and CTD-ILD). Interestingly, in the IPAF group, we observed a negative correlation between KL-6 concentration and all the analysed PFTs (FVC%, TLCO%, and 6MWT distance) and SpO2 measurement at baseline. These results are consistent with the available literature. In the study by Xue et al., a negative correlation between KL-6 concentration and TLCO% and FVC% in patients with IPAF was described, while the study by Wang et al. only demonstrated a correlation with TLCO% [9,10]. Notably, there has been no research in the IPAF group on the other functional parameters (including 6MWT distance) and SpO2. Moreover, SP-D in the IPAF group correlated with the 6MWT distance and SpO2 at baseline, which is a unique observation not yet analysed in the literature. A strong correlation of the serum KL-6 levels with the ventilatory parameters is mainly described in another ILD—idiopathic pulmonary fibrosis (IPF) [15,16]. Our results may indicate a common pathobiological substrate in IPF and IPAF, where pulmonary fibrosis seems to be the most pronounced component.

The analysis of the CTD-ILD group yielded surprising results. In these patients, circulating KL-6 and SP-D did not serve as reliable biomarkers for lung impairment. No correlations were found between their levels and FVC%, TLCO%, and 6MWT distance. Instead, molecule levels showed a positive correlation with cell count in BAL (KL-6 and SP-D) and an inverse correlation with the time since symptom onset (KL-6). This indicates that in this group, the levels of the studied biomarkers reflect the intensity of the inflammatory process in the respiratory tract, rather than lung function impairment. Our results differ from those presented by some researchers, who report that in patients with CTD-ILD, KL-6 levels may negatively correlate with the FVC% and TLCO% values. The evaluation of SP-D correlation with PFT results, according to the available literature, remains inconclusive, and only some studies describe any significant relationship between these parameters [17,18]. The discrepancies between our study results and those of other publications, which demonstrated a correlation with PFTs, may arise from the small sample size of our study and the heterogeneity observed among connective tissue diseases (differences between individual disease entities, e.g., SSc, IIM, and RA). Consequently, the study results can vary depending on the proportions of different patient groups included in the studies. However, in the study by Hant et al., the authors described that in patients with SSc-ILD without alveolitis (defined as a BAL fluid sample with ≥3% neutrophils, ≥2% eosinophils or both on a cell differential count, or any ground-glass opacity on HRCT), no correlation was observed between the KL-6 and SP-D levels and FVC% and TLCO% [19]. Another significant factor is that our study included both patients with radiologic patterns indicative of lung fibrosis (UIP and fNSIP) and those with the predominance of inflammation (e.g., organising pneumonia—OP and lymphoid interstitial pneumonia—LIP).

When analysing the entire autoimmune-related ILD group (IPAF and CTD-ILD), we found that SP-D concentration correlated inversely and TGF-β1 positively with TLCO%. Additionally, the levels of KL-6 and SP-D show a negative correlation with the 6MWT distance.

The molecules assessed in our study are poor predictors of short-term prognosis. Their baseline levels did not predict changes in FVC% or TLCO% during a one-year follow-up period in any group of patients. However, it is noteworthy that the KL-6 levels correlated with changes in the 6MWT distance in both the CTD-ILD group and across the broader category of autoimmune-related ILD (IPAF and CTD-ILD). To the best of our knowledge, there are no existing studies on the usefulness of this biomarker in assessing the baseline 6MWT distance and predicting changes in this parameter over time.

This may be due to the fact that the 6MWT test should ideally be performed consistently by the same professionals under the same conditions, which can be challenging to achieve in clinical practice.

While numerous studies indicate that baseline concentrations of biomarkers such as KL-6 and SP-D are not useful as short-term predictors of disease progression for patients with IPAF or CTD-ILD, increases in these biomarker levels are associated with decreases in PFT measurements (primarily FVC% and TLCO%) over time, suggesting their potential predictive value for long-term disease progression [9,20,21].

Our study has certain limitations. First, this is a single-centre study, which may limit the generalizability of the findings and introduce selection bias. However, most of the studies cited in this article are also single-centre studies [9,10,11,13,14,20]. Second, the study groups were relatively small, but given the rarity of the condition, particularly IPAF, the sample sizes are comparable to those in other studies [13,14,20]. Third, the ILD patients included in the research presented various stages of the disease, ranging from mild and moderate to severe lung function impairment.

## 4. Materials and Methods

### 4.1. Study Design and Methods

The single-centre study was conducted at the Department of Pneumology of the Medical University of Lodz. At a multidisciplinary meeting involving a pulmonologist, radiologist, and rheumatologist, we prospectively identified patients classified as IPAF according to the ERS/ATS research statement between March 2021 and November 2022 [1]. During the same period, we recruited the control groups: (1) CTD-ILD and (2) CTD without ILD. We diagnosed CTD based on the EULAR/ACR classification criteria [22,23,24,25,26]. The exclusion criteria included other respiratory diseases, any history of neoplastic processes, age of under 18 years, or missing relevant data.

All study participants underwent clinical assessments by a multidisciplinary team (MDT), including pulmonologists, rheumatologists, and radiologists, at baseline and after 12 months. The evaluations included medical history, physical examination, baseline peripheral venous blood sampling, and pulmonary function tests: spirometry, TLCO, and 6MWT. Antibodies were assessed both at baseline and after 12 months to evaluate the fulfilment of the IPAF criteria and potential seroconversion. Dyspnea was assessed using the Modified Medical Research Council (mMRC) scale. Time since symptom onset was assessed in years. Initial symptoms included both respiratory (shortness of breath, cough) and systemic (e.g., joint pain, fever) manifestations, except for Raynaud’s phenomenon. HRCT was performed at two time points, except for the control group CTD without ILD (performed only at baseline).

The study reports the initial preliminary findings of a broader investigation approved by the Ethics Committee of the Medical University in Lodz (RNN/43/21/KE). All patients signed a written consent form.

### 4.2. Pulmonary Function Tests

Spirometry and TLCO measurements were conducted using the Lungtest 1000 system (MES, Cracow, Poland) in accordance with ATS and ERS standards [27,28]. Recorded parameters included forced expiratory volume in 1 s (FEV_1_), forced vital capacity (FVC), and TLCO measurements (adjusted for haemoglobin concentration). The results of the PFTs were expressed as a percentage of the predicted values (% pred.) using reference values from the Global Lung Function Initiative (GLI). Then, 6MWT was performed at each time point by the same person in the same place according to the current guidelines [29,30].

### 4.3. HRCT Evaluation

Chest high-resolution computed tomographies were reviewed and scored independently by 2 experienced pulmonologists (W.P. and E.M.). The quantitative assessment (%ILD) was performed according to the Polish Respiratory Society experts’ recommendations [31]. Radiological patterns were described according to the current ATS/ERS guidelines [32,33].

### 4.4. Processing of Bronchoalveolar Lavage Fluid

Bronchoscopy with bronchoalveolar lavage fluid processing was carried out for diagnostic purposes according to the recommendations of the Polish Respiratory Society [34,35].

### 4.5. Assessment of ILD Short-Term Progression According to Different Definitions

Progression was defined based on four criteria: progressive pulmonary fibrosis (PPF, as per the 2022 ATS/ERS/JRS/ALAT guidelines), progressive fibrosing interstitial lung disease (PF-ILD, based on the INBUILD criteria), the Polish Respiratory Society guidelines, and an FVC decline of ≤10% [33,35,36]. Each criterion was analysed independently.

### 4.6. Serum Biomarker Measurements

Blood samples were obtained by venipuncture and stored at −80 °C for further analysis. The serum KL-6, SP-D, and TGF-β1 levels were evaluated by enzyme-linked immunosorbent assay (ELISA) kits following the manufacturer’s instructions. The measurement was performed using commercially available ELISA kits (KL-6: Cloud Clone Corp., Katy, TX, USA, Lot No: L210506932; SP-D: BioVendor-Laboratorni medicina a.s., Brno, Czech Republic, Lot No: E-21-023; TGF-β1: Cloud-Clone Corp., Katy, TX, USA, Lot No: 650.010.096).

### 4.7. Statistical Analysis

Continuous data were presented as means with standard deviations (SDs) or medians with interquartile ranges (IQRs), depending on the data distribution. Between-group comparisons were conducted using the unpaired Student’s *t*-test, Welch’s *t*-test, Wilcoxon rank-sum test, Kruskal–Wallis test, or ANOVA, based on the normality of the data and variance homogeneity. For the Kruskal–Wallis test and ANOVA, post hoc comparisons were performed using the Bonferroni method. Categorical data were analysed using Pearson’s Chi-squared test or Fisher’s Exact Test. Correlation analysis was performed using Spearman’s rho test and presented as a heatmap. Missing data were not imputed in the analysis. Statistical analysis was performed using R software version 4.3.3 for macOS.

## 5. Conclusions

Our study showed that the concentrations of KL-6, SP-D, and TGF-β1 are higher in patients with autoimmune-related ILD than in CTD patients without ILD, indicating their potential as biomarkers for the presence of lung fibrosis in these conditions. KL-6 and SP-D levels correlate with HRCT-based ILD assessments, suggesting some predictive value for fibrosis severity, though their utility in short-term prognosis is limited. Associations between biomarker levels and clinical features vary by group; in IPAF, KL-6 and SP-D correlate inversely with baseline PFTs and exercise capacity, while in CTD-ILD, they correlate with BAL cellularity and disease duration. Further studies with larger cohorts are required to confirm these preliminary findings and establish the specific diagnostic and monitoring roles of these biomarkers in autoimmune-related ILD.

## Figures and Tables

**Table 1:** Baseline characteristics of the included patients.

	IPAF	CTD-ILD	CTD Without ILD	*p*
n	24	21	23	
Age, years, median [IQR]	61 [51.75–67.50]	61 [51–67]	58 [48.5–65]	0.61
Female sex, n (%)	18 (75)	16 (80)	20 (87)	0.59
History of smoking, n (%)	9 (40.9)	7 (36.8)	10 (45.5)	0.85
mMRC, median [IQR]	2 [1,2]	1 [1,2]	0 [0–0]	**<0.0001**
Radiological pattern, n (%)	UIP: 6 (25) NSIP: 11 (46) Other: 7 (29)	UIP: 7 (33) NSIP: 10 (48) Other: 4 (19)	-	0.69
**PFTs**				
FVC%, mean (SD)	69 (20.78)	62 (16.03)	90 (10.79)	**<0.0001**
TLCO%, mean (SD)	59.57 (19.64)	65.18 (17.28)	96.14 (15.76)	**<0.0001**
6MWT distance, m, mean (SD)	408.1 (117.17)	389.6 (119.01)	481.2 (101.83)	**0.03**

IPAF—interstitial pneumonia with autoimmune features, CTD—connective tissue disease, ILD—interstitial lung disease, IQR—interquartile range, SD—standard deviation, mMRC—Modified Medical Research Council, PFT—pulmonary function test, FVC—forced vital capacity, TLCO—lung transfer factor for carbon monoxide adjusted for haemoglobin, 6MWT—six-minute walk test, UIP—usual interstitial pneumonia, and NSIP—non-specific interstitial pneumonia.

**Table 2:** Biomarker concentrations across patient groups.

	IPAF	CTD-ILD	CTD Without ILD	*p*
KL-6, U/mL, median, [IQR]	1508 [851.5–2307]	1113 [787–1854]	312 [273.5–399]	**<0.0001**
SP-D, ng/mL median, [IQR]	52.2 [38.4–95.9]	59.6 [46.5–89.5]	34.9 [20–47.6]	**0.0005**
TGF-β1, pg/mL, median, [IQR]	151.3 [73.2–212.4]	103.5 [66.4–193.8]	35.2 [21.3–48.9]	**0.0001**

IPAF—interstitial pneumonia with autoimmune features, CTD—connective tissue disease, ILD—interstitial lung disease, IQR—interquartile range, KL-6—Krebs von den Lungen-6, SP-D—surfactant protein D, and TGF-β1—transforming growth factor β.

## Data Availability

The data presented in this study are available from the corresponding author upon reasonable request.

## Supplementary Materials

The following supporting information can be downloaded at: https://www.mdpi.com/article/10.3390/ijms26031091/s1.
