# Supplementary material for: Serum KL-6 and SP-D: Markers of Lung Function in Autoimmune-Related Interstitial Lung Diseases
Source: Int J Mol Sci. 2025 Jan 27;26(3):1091. doi: 10.3390/ijms26031091 (PMC11817276; doi:10.3390/ijms26031091)
Supplement: Supplementary file 1 [file ijms-26-01091-s001.zip › ijms-3370685-supplementary.pdf]

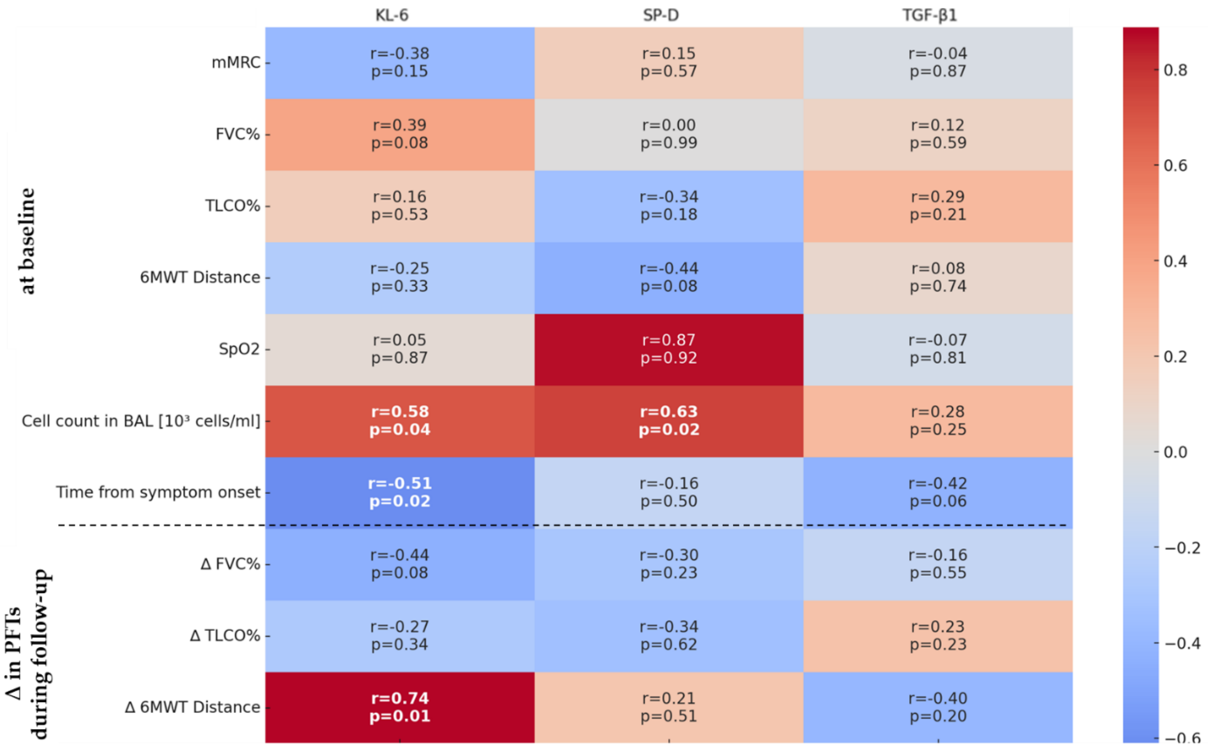

Supplementary Figure S1. The correlation heatmaps between serum biomarkers KL6, SP-D, TGF-β1 and lung function or clinical indicators in the CTD-ILD group. The heatmap colour corresponds to the r-value (−0.6 to +0.8). The upper row in each table square denotes the r-value of the Spearman's rank correlation, and the lower row denotes its p-value. The bolded values are statistically significant. CTD – connective tissue disease, ILD – interstitial lung disease, mMRC—modified Medical Research Council, FVC—forced vital capacity, TLCO - lung transfer factor for carbon monoxide adjusted for haemoglobin, 6 MWT—six-minute walk test, SpO2 – peripheral capillary oxygen saturation, as measured by pulse oximetry, BAL - bronchoalveolar lavage, PFT – pulmonary function test.

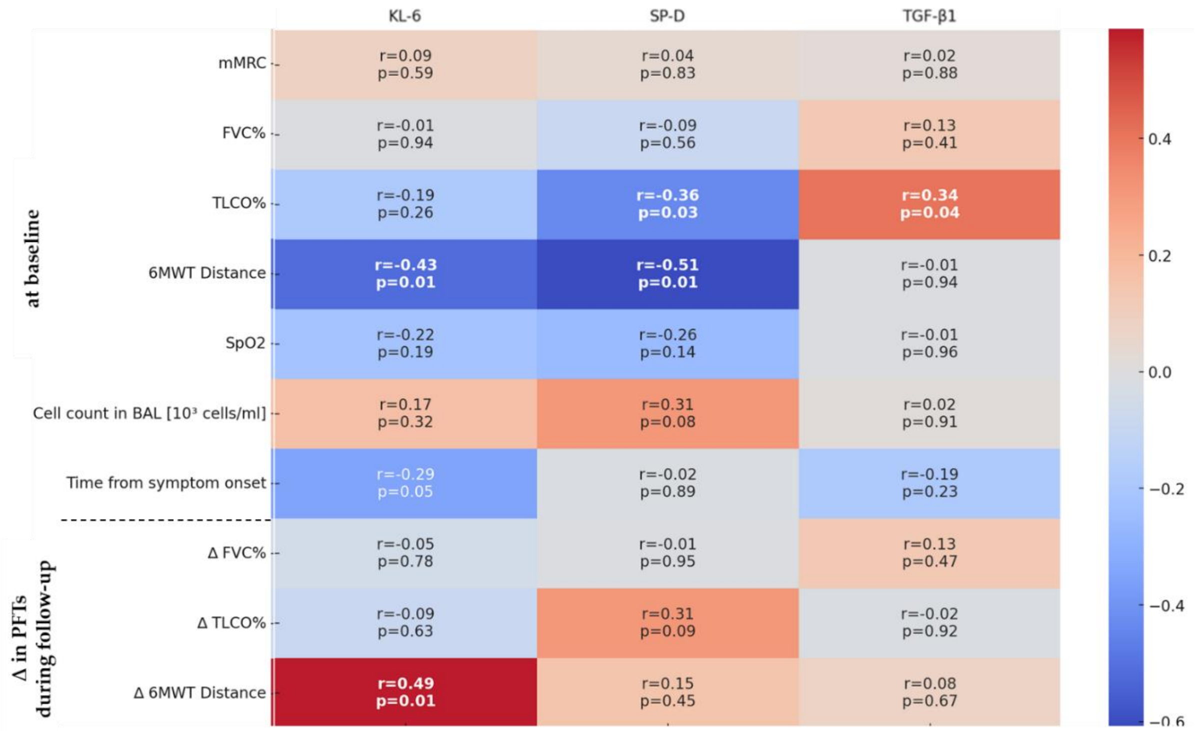

Supplementary Figure S2. The correlation heatmaps between serum biomarkers KL6, SP-D, TGF-β1 and lung function or clinical indicators in patients with autoimmune-mediated ILD (IPAF and CTD-ILD). The heatmap colour corresponds to the r-value (−0.6 to +0.6). The upper row in each table square denotes the r-value of the Spearman's rank correlation, and the lower row denotes its p-value. The bolded values are statistically significant. IPAF – interstitial pneumonia with autoimmune features, CTD – connective tissue disease, ILD – interstitial lung disease, mMRC—modified Medical Research Council, FVC—forced vital capacity, TLCO - lung transfer factor for carbon monoxide adjusted for haemoglobin, 6 MWT—six-minute walk test, SpO2 – peripheral capillary oxygen saturation, as measured by pulse oximetry, BAL - bronchoalveolar lavage, PFT – pulmonary function test.
